# CO_2_ Efflux from Cleared Mangrove Peat

**DOI:** 10.1371/journal.pone.0021279

**Published:** 2011-06-29

**Authors:** Catherine E. Lovelock, Roger W. Ruess, Ilka C. Feller

**Affiliations:** 1 School of Biological Sciences, The University of Queensland, St Lucia, Queensland, Australia; 2 Institute of Arctic Biology, University of Alaska Fairbanks, Fairbanks, Alaska, United States of America; 3 Smithsonian Environmental Research Center, Edgewater, Maryland, United States of America; National Institute of Water & Atmospheric Research, New Zealand

## Abstract

**Background:**

CO_2_ emissions from cleared mangrove areas may be substantial, increasing the costs of continued losses of these ecosystems, particularly in mangroves that have highly organic soils.

**Methodology/Principal Findings:**

We measured CO_2_ efflux from mangrove soils that had been cleared for up to 20 years on the islands of Twin Cays, Belize. We also disturbed these cleared peat soils to assess what disturbance of soils after clearing may have on CO_2_ efflux. CO_2_ efflux from soils declines from time of clearing from ∼10 600 tonnes km^−2^ year^−1^ in the first year to 3000 tonnes km^2^ year^−1^ after 20 years since clearing. Disturbing peat leads to short term increases in CO_2_ efflux (27 umol m^−2^ s^−1^), but this had returned to baseline levels within 2 days.

**Conclusions/Significance:**

Deforesting mangroves that grow on peat soils results in CO_2_ emissions that are comparable to rates estimated for peat collapse in other tropical ecosystems. Preventing deforestation presents an opportunity for countries to benefit from carbon payments for preservation of threatened carbon stocks.

## Introduction

Mangroves are being cleared at a rapid rate, exceeding that of tropical forests [1], [2]. Clearing of above-ground biomass in mangrove forests results in changes in ecosystem processes [3] and losses of ecosystem services, including fisheries and storm protection [4], [5]. Additionally, clearing of forests reduces carbon sequestration and may lead to CO_2_ emissions due to loss of aboveground carbon stocks and increased rates of soil decomposition [6]. In terrestrial ecosystems land-use change is one of the major sources of CO_2_ emissions above the burning of fossil fuels [7]. In the tropics clearing of rainforests has led to high levels of CO_2_ emissions [8] which have made these forests particularly valuable for conservation schemes developed to reduce emissions from deforestation and forest degradation, and to enhance carbon storage (REDD and REDD+) [9], [10], [11]. Similar schemes are proposed for carbon rich marine ecosystems, including mangroves, but there are many uncertainties around factors influencing carbon sequestration and carbon stocks in these coastal systems [12], [13].

There are few estimates of ecosystem carbon stocks in mangroves [14], [15]. The few that are available indicate a large proportion of carbon is in soils [14], [15], [16]. Carbon stocks in mangrove soils can be extremely high at some sites, as they contain accumulated peat (>20% carbon) derived mainly from roots as sea level has risen in the last interglacial period and anoxic conditions have slowed decomposition [17], [18]. The high levels of carbon in mangrove soils, the potential oxidation of peat deposits with land use change [6] indicate that once cleared mangrove forests on peat soils may become significant sources of CO_2_.

In terrestrial tropical forest settings, clearing and draining of peat soils results in oxidation of carbon leading to peat collapse and the emission of CO_2_ and other greenhouse gases [11]. Peat collapse and CO_2_ emissions from cleared peat lands correlate with the level of the water table, increasing with the lowering of the water table and thus the exposure of peat to aerobic conditions [11], [19], [20]. Similarly, clearing of mangrove forests could result in significant CO_2_ emissions due to oxidation of C in mangrove peat. In mangrove ecosystems that have been damaged by hurricanes peat collapse has been observed [21]. Additional oxidation may occur if peat is disturbed and contact with air is increased, as would be the case when shrimp ponds are constructed in peat soils and peat is pushed up on to banks or levees. The increase in CO_2_ emissions with clearing of mangroves may be a major cost of disturbance of mangrove world-wide, and thus may contribute to the case for strengthening protection of these ecosystems through abating CO_2_ emissions [12], [22].

In this study we measured CO_2_ emissions from mangrove peat in Belize that had been cleared of vegetation over the last 2 decades in anticipation of tourism development. This mangrove peat at the site has a carbon (C) concentration of approximately 300 mg C g^−1^ [23]. We used a chronosequence of clearing to assess the potential change in CO_2_ efflux over time since disturbance. Additionally we experimentally disturbed cleared peat to assess the potential enhancements in CO_2_ efflux through increasing contact with air.

## Results

**Figure 1:**
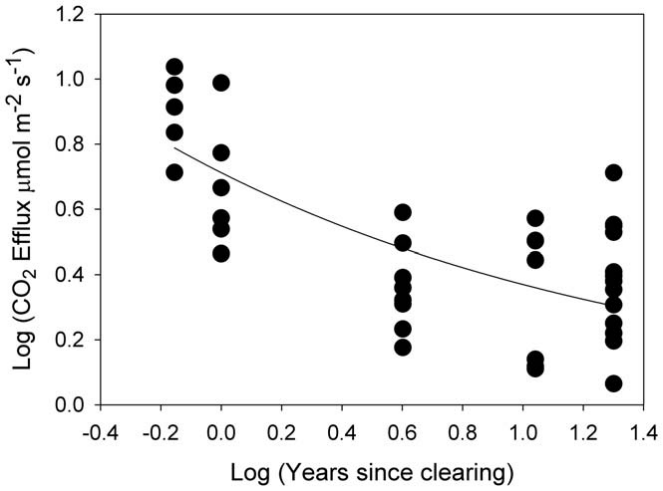
Variation in CO_2_ efflux from peat soils over the time since the mangrove forest was cleared from Twin Cays Belize. The fitted line is of the form: Log CO_2_ Efflux  =  a x exp (-b x time) where a = 0.712 and b = 0.656; R^2^ = 0.51. The model is significant: F_1,30_ = 40.4988, P<0.0001.

**Figure 2:**
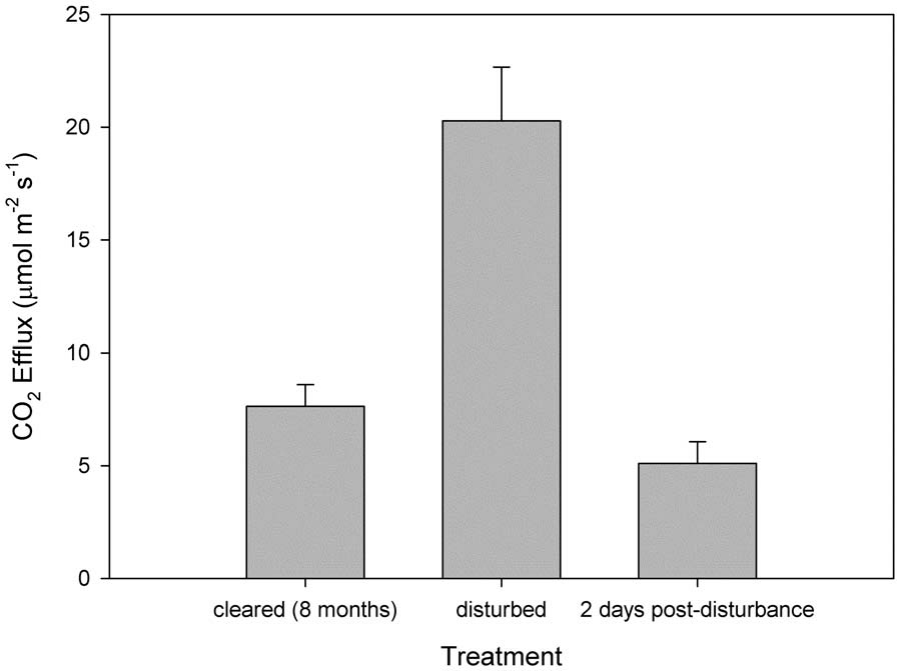
CO_2_ efflux from peat soils that were cleared of forest (cleared 8 months) where peat was disturbed by cutting blocks from the soils (disturbed) and two days after the blocks of peat were cut (2 days post-disturbance). There was a significant effect of the disturbance treatment (F_2,15_ = 25.37, P<0.0001) but after two days there was no significant difference in soil CO_2_ efflux between disturbed and undisturbed samples.

Over our chronosequence of sites representing time since clearing of mangroves, CO_2_ efflux declined logarithmically with time, from 7.6 to 2.1 µmol m^−2^ s^−1^ over 20 years (Figure 1, F_1, 30_ = 40.50, P<0.0001). Soil temperature varied during the measurements, but there was no significant correlation between CO_2_ efflux and soil temperature. At 4 years after clearing, CO_2_ efflux had reached a relatively constant level of approximately 2 µmol m^−2^ s^−1^. Extrapolation of CO_2_ efflux rates to annual CO_2_ loss indicates that CO_2_ emissions from cleared peat would be ∼10 600 tonnes km^−2^ year^−1^ in the first year after clearing, falling to ∼2900 tonnes CO_2_ km^−2^ year^−1^ (Table 1). Higher rates of CO_2_ efflux were observed with acute disturbance of the peat, reaching a mean of 27 µmol m^−2^ s^−1^ when blocks of peat were cut from the soil (Figure 2, F_2,15_ = 25.37, P<0.0001). However this increase was transitory, as CO_2_ efflux had returned to ambient levels within 2 days of disturbance.

**Table 1:** Estimates of CO_2_ efflux from modified mangrove and other habitats with peat soils.

Habitat	Modification	CO_2_ efflux tonnes km^−2^ year^−1^	Method	Reference
Mangrove, Belize	Cleared	2900	CO_2_ efflux	THIS STUDY
Mangrove, Honduras	Forest damaged by hurricane	1500	Inferred from peat collapse	Cahoon et al. 2003
Mangrove, Australia	Shrimp pond	1750 (220–5000)	CO_2_ efflux	Burford and Longmore 2001
Rainforest, Indonesia	Drained for agriculture	3200	Inferred from peat collapse and measured as CO_2_ efflux	Couwenburg et al. 2010 and references therein
Tundra, Alaska	Thawed (vegetation intact)	150–430	Net CO_2_ exchange	Schuur et al. 2009

## Discussion

Based on short term measurements of CO_2_ efflux from the soil surface of cleared mangrove forests, we found that CO_2_ efflux is substantial, estimated to be approximately 2900 tonnes km^−2^ year^−1^ (Table 1). This value is similar to CO_2_ losses estimated for collapsing terrestrial peat soils in Indonesia [11], similar to that which can be estimated from peat collapse (losses in elevation) after hurricane damage in mangroves in Honduras [21], and greater than estimates of CO_2_ emissions with permafrost thaw and decomposition of tundra peat [24]. In contrast, intact mangrove forests absorb approximately 5000 tonnes CO_2_ km^−2^ year^−1^ of which only ∼20% is respired as CO_2_ [14], [15]. Carbon export from mangroves to adjacent systems (which could be up to 70% of total production) may potentially contribute to CO_2_ emissions, but also support secondary production [14], [15]. Clearing mangroves from peat soils will clearly be unfavourable for regional and global carbon budgets [6] as well as reducing other ecosystem services offered by mangroves [5].

While CO_2_ efflux from intact forest soils is strongly associated with root respiration [25], CO_2_ efflux from cleared and disturbed mangrove soils reflects microbial degradation of organic matter within soils [15]. The large, but transient increase in CO_2_ efflux with disturbance of the peat (Figure 2) probably reflects oxidation of relative labile fractions (e.g. sugars and phenols) as they are exposed to enhanced oxygen concentrations [26]. However, this fraction is rapidly depleted before relatively slower decomposition of refractory pools (e.g. lignin) dominates CO_2_ efflux. Short term high levels of CO_2_ efflux from soil directly after clearing (Figure 1, 8 months) or from disturbing the peat are not included in our annual estimate of CO_2_ emissions but may contribute a significant proportion to total emissions.

Once cleared, mangroves are often converted to shrimp ponds [1], [2]. Rates of CO_2_ emissions from cleared mangroves are within the same range as those measured from shrimp ponds [27]. Thus, once established this alternative land-use, unlike conversion to agriculture [28] does not mitigate CO_2_ emissions from clearing mangroves. Additionally, aquaculture and agriculture often increase nutrient availability of coastal waters [29]. Mangrove peat collapse has been observed to be enhanced by addition of nitrogen due to increases in decomposition and compaction [18]. Thus, increasing levels of nutrients in cleared mangrove areas may contribute to loss of habitat and possibly to increased CO_2_ emissions associated with decomposition of peat.

Approximately half of Caribbean mangrove forests are anticipated to be growing on carbon rich peat soils [30]. The proportion of mangrove forests on peat soils is not known for the Indo-Pacific region and Africa, but could be substantial, particularly if mangrove peat is associated with upland peat forest soils which are common in the Indo-Pacific region [31]. The documentation of acid sulphate soils in shrimp farm developments from South East Asia and elsewhere [32] also indicates the presence of high concentrations of organic matter in many mangrove soils that have already been cleared for aquaculture. CO_2_ emissions from cleared mangroves growing on mineral soils have not been assessed, but are needed in conjunction with improved soil mapping of soil carbon stocks within mangrove forest soils in order to estimate the global effects of clearing mangroves on CO_2_ emissions.

Our annual estimate of CO_2_ emissions of 2900 tonnes km^−2^ year^−1^ may be improved through measurement of CO_2_ efflux over seasons which vary in tidal height, temperature and rainfall, however the timing of our measurements have probably lead to a underestimate of CO_2_ emissions. Our measurements were made in the winter months in Belize when temperatures are relatively low and may limit bacterial activity. Although tidal variation is low in Belize (∼0.5 m) in the winter months tides are higher than in summer [33] and thus we may have underestimated CO_2_ flux compared to periods when tides are lower and peat maybe exposed to air at greater depth in the soil. Increases in sea level may also influence CO_2_ emissions from cleared forest soils, changing oxidation status and potentially altering decomposition processes [26].

We conclude that the clearing of mangroves and the use of mangrove peat soils for alternative uses (e.g. cleared, shrimp ponds) results in increases in CO_2_ emissions, in addition to resulting in losses in other ecosystem functions including fisheries and coastal protection. Incentive payments for maintaining intact forests, thus avoiding carbon emissions, as proposed by REDD and REDD+ [9], would be beneficial for conservation of mangroves in the tropics. There are significant gaps in our knowledge in: 1) the global extent of carbon currently stored in peat and mineral soils in mangrove forests, 2) the rate of CO_2_ emissions from clearing mangroves growing on mineral soils, 3) the spatial and temporal variation in CO_2_ emissions from cleared mangrove forests and alternative land-uses, and 4) the loss of carbon as dissolved organic and inorganic forms of carbon from intact and disturbed forest systems [12], [14], [15]. Filling these knowledge gaps will improve arguments for conservation of mangroves based on carbon stocks and sequestration.

## Materials and Methods

### Study site

This study was conducted at Twin Cays, a peat-based, 92-ha archipelago of intertidal mangrove islands in a carbonate setting, just inside the crest of the Mesoamerican Barrier Reef System of central Belize, 12 km off shore (16°50′N, 88°06′W). These islands receive no terrigenous inputs of freshwater or sediments. Mangrove islands in this part of the reef, which originated approximately 8000 yr B.P. on a limestone base formed by a Pleistocene patch reef, have an underlying peat deposit ∼7–10 m thick and have been mangrove communities throughout the Holocene [18]. Mangrove forests are dominated by *Rhizophora mangle* the roots of which the peat is derived [18]. Since 1980, this group of islands has been the primary study site for the Smithsonian Institution's National Museum of Natural History Field Station on nearby Carrie Bow Cay [34].

Illegal clearing of mangroves has occurred on Twin Cays over the last 20 years, primarily for housing and prospective tourism developments. Multiple clearing events allowed us to measure CO_2_ efflux over soils that have been cleared of vegetation over 20 years. We measured CO_2_ efflux from 3–5 ha patches that had been cleared for durations of 8 months, 12 months, 4 years, 11 years and 20 years. We measured efflux at 6–12 locations within each aged clearing.

In order to test whether disturbance of the peat increased soil CO_2_ efflux we cut blocks of peat from the area that had been cleared 8 months previously. Six replicate blocks approximately 30×30×30 cm were cut with a shovel and placed on the soil surface. We measured CO_2_ efflux from the soil, from the peat blocks directly after cutting them from the peat and then again after 2 days.

CO_2_ efflux from soils was measured using a LiCor 6400 portable photosynthesis system configured with the LiCor Soil Respiration chamber (LiCor Corp, Lincoln, NE, USA). The chamber was set to penetrate 5 mm into the soil. Settings for measurement were determined at each site following the procedure described by the manufacturer. Soil temperature was measured at 2 cm depth simultaneously with CO_2_ efflux. Soil temperatures varied from 28 to 34 C during the measurements. Measurements were made in February of 2004 and January of 2007.

### Data analysis

Differences in soil CO_2_ flux over time among areas of differing time since clearing were assessed using linear models where time was considered a random, continuous variable in the model. Changes in CO_2_ efflux with disturbance of peat was assessed using repeated measures ANOVA. Scaling instantaneous CO_2_ efflux data was done by simply multiplying CO_2_ efflux (µmol m^−2^ s^−1^) by time to give tonnes CO_2_ km^−2^ year^−1^.
